# Immunotherapy-related adverse events (irAEs): extraction from FDA drug labels and comparative analysis

**DOI:** 10.1093/jamiaopen/ooy045

**Published:** 2018-10-15

**Authors:** QuanQiu Wang, Rong Xu

**Affiliations:** Department of Population and Quantitative Health Sciences, School of Medicine, Case Western Reserve University, Cleveland, Ohio, USA

**Keywords:** cancer immunotherapy, checkpoint inhibitors, immunotherapy-related adverse events, information extraction, data analysis

## Abstract

**Objectives:**

Immune checkpoint inhibitors (ICIs) have dramatically improved outcomes in cancer patients. However, ICIs are associated with significant immune-related adverse events (irAEs) and the underlying biological mechanisms are not well-understood. To ensure safe cancer treatment, research efforts are needed to comprehensively detect and understand irAEs.

**Materials and methods:**

We manually extracted and standardized irAEs from The U.S Food and Drug Administration (FDA) drug labels for six FDA-approved ICIs. We compared irAE profile similarities among ICIs and 1507 FDA-approved non-ICI drugs. We investigated how irAEs have differential effects on human organs by classifying irAEs based on their targeted organ systems. Finally, we identified broad-spectrum (nontarget-specific) and narrow-spectrum (target-specific) irAEs.

**Results:**

A total of 893 irAEs were extracted. 31.4% irAEs were shared among ICIs as compared to the 8.0% between ICIs and non-ICI drugs. irAEs were resulted from both on- and off-target effects: irAE profiles were more similar for ICIs with same target than different targets, demonstrating the on-target effects; irAE profile similarity for ICIs with the same target is less than 50%, demonstrating unknown off-target effects. ICIs significantly target many organ systems, including endocrine system (3.4-fold enrichment), metabolism (3.7-fold enrichment), immune system (3.6-fold enrichment), and autoimmune system (4.8-fold enrichment). We identified 21 broad-spectrum irAEs shared among all ICIs, 20 irAEs specific for PD-L1/PD-1 inhibition, and 28 irAEs specific for CTLA-4 inhibition.

**Discussion and conclusion:**

Our study presents the first effort toward building a standardized database of irAEs. The extracted irAEs can serve as the gold standard for automatic irAE extractions from other data resources and set a foundation to understand biological mechanisms of irAEs.

## INTRODUCTION

Paradigm-shifting checkpoint inhibition immunotherapies have transformed outcomes for patients with melanoma and other metastatic cancers.^1^ Immune checkpoint inhibitors (ICIs) kill tumor cells by targeting immunosuppressive molecules expressed on immune cells, including cytotoxic T-lymphocyte-associated antigen 4 (CTLA-4), programmed cell death protein (PD-1), and its ligand PD-L1.^2^ Currently there are six FDA-approved ICIs that target CTLA-4 (ipilimumab), PD-L1 (atezolizumab, avelumab, and durvalumab), and PD-1 (nivolumab and pembrolizumab).^3^ Many others are being actively tested in thousands of clinical trials.^4^ ICIs are promising new generation of cancer treatments; however, they are expensive and often associated with immunetherapy-related adverse events (irAEs) across organ systems, ranging from mild to severe and life threatening^5^ (Table 1).

**Table 1.** Six FDA-approved immune checkpoint inhibitors

Name	Target	First approval indication	Year	Cost/year	irAEs
Atezolizumab	PD-L1	Non-small cell lung cancer	2016	$150 000	Pneumonitis, endocrinopathy, myocarditis, hepatitis, colitis
Avelumab		Merkel cell carcinoma	2017	$156 000	Pneumonitis, hepatitis, colitis, endocrinopathy, nephritis, arthritis
Durvalumab		Urothelial carcinoma	2017	$180 000	Hepatitis, dermatitis, endocrinopathy, colitis, pneumonitis
Ipilimumab	CTLA-4	Melanoma	2011	$158 252	Enterocolitis, hepatitis, dermatitis, neuropathy, endocrinopathy
Nivolumab	PD-1	Melanoma	2014	$103 220	Encephalitis, pneumonitis, colitis, hepatitis, endocrinopathy, nephritis
Pembrolizumab		melanoma	2014	$150 000	Pneumonitis, colitis, hepatitis, nephritis, encephalitis, endocrinopathy

Information were manually extracted from web-search for authoritative websites including Drugs.com^6^ and DailyMed^7^

The population of cancer patient taking ICIs is large and fast growing^8^; however, the biological mechanisms by which ICIs exert their adverse effects on different organs are not well-understood. To ensure safe personalized cancer treatment, research efforts are needed to comprehensively curate, characterize, and understand the biological basis of irAEs. The knowledge of irAEs are buried in different resources in different format, including The U.S Food and Drug Administration (FDA) drug labels, published biomedical literature, electronic patient health records (EHRs), FDA postmarket drug safety surveillance system (FAERS), among others. Currently, there exist no structured data resources of irAEs. Side Effect Resource (SIDER) is a structured database of drug-side effect associations extracted from FDA drug labels using text mining techniques.^9^ However, the most updated version of SIDER (SIDER4.1) contains none of the FDA-approved ICIs. Our extensive prior works in developing natural language processing and text mining techniques to extract drug-side effect (SE) relationships from free-text documents demonstrated that automatic drug-SE relationship extractions are challenging and often have limited precision and recall.^10–17^

In this study, we manually extracted irAEs for six FDA-approved ICIs from their FDA drug labels, standardized and mapped the extracted terms to the Medical Dictionary for Regulatory Activities (MedDRA), a widely used terminology for encoding drug side effects,^18^ and performed comparative analysis of extracted irAE profiles. Our goals are 3-fold. First, the manually irAEs can serve as the gold standard for automatic irAE extractions from other data resources such as biomedical literature, EHRs, and FAERS. Second, the large number of irAEs extracted from FDA drug labels can advance our knowledge concerning the scope of irAEs in cancer patients. Third, the extracted irAEs, when combined with vast amounts of genetic and genomics data of drugs and diseases, can set the foundation to develop computational approaches to understand biological mechanisms that contribute to irAEs. To the best our knowledge, our study presents the first effort to build a highly accurate, standardized database of irAEs associated with ICIs and to perform comparative analysis to characterize and understand extracted irAEs.

## METHODS

### Manual extraction of irAEs from FDA drug labels and mapping them to MedDRA preferred terms

#### Extract irAEs from FDA drug labels

Currently there are six FDA-approved ICIs: atezolizumab (PD-L1 inhibitor), avelumab (PD-L1 inhibitor), durvalumab (PD-L1 inhibitor), ipilimumab (CTLA-4 inhibitor), nivolumab (PD-1 inhibitor), and pembrolizumab (PD-1 inhibitor).^3^ For each drug, we obtained its drug label from DailyMed^7^ (data accessed in October 2017). DailyMed is the official provider of FDA label information (package inserts) and provides trustworthy, comprehensive, and up-to-date information about marketed drugs in the United States. We manually extracted adverse reactions from the following sections: “BOXED WARNING” (if applicable), “WARNINGS AND PRECAUTIONS,” and “ADVERSE REACTIONS.” Adverse reaction information from both free text and embedded tables were manually extracted.

#### Mapping and standardization

We then mapped extracted irAE terms to terms in MedDRA, an international medical terminology of drug side effects used by FDA and other regulatory authorities in the pharmaceutical industry during the regulatory process.^18^ The most updated version of MedDRA includes 70 000 “Lowest Level Terms” (LLTs) and each LLT is linked to one unique “Preferred Terms” (PTs). During our mapping process, we found that the majority (95%) of extracted irAE terms could be mapped to LLT terms. We then unified all mapped LLT terms to their PTs. For example, the LLTs “fraility,” “energy decreased,” “weakness,” “debility,” “loss of energy,” and “physical deconditioning” were mapped to the single PT term “asthenia.”

### Calculate pairwise AE profile similarities among six ICIs and 1507 FDA-approved non-ICI drugs

We calculated the pairwise irAE profile similarities among ICIs using extracted irAEs. We also calculated the similarities between ICIs and 1507 FDA-approved drugs using extracted irAEs and drug-AE associations from SIDER.^9^ Currently, SIDER contains 167 411 drug-AE pairs (149 159 drug-PT pairs) for 1507 drugs and 6088 AEs (4231 PTs) (data accessed in 10/2017). Note that even the most updated version SIDER4.1 does not contain any of the six FDA-approved ICIs. We used the 149 159 drug-PT pairs to construct AE profiles for 1507 FDA-approved drugs. We used the Jaccard similarity coefficient^19^ for comparing the similarity of AE profiles of any two drugs. The Jaccard similarity coefficient of two AE profiles is defined as the size of the intersection divided by the size of the union of two AE profiles: $J(A,B)=\frac{\left|A\cap B\right|}{\left|A\cup B\right|}=\frac{\left|A\cap B\right|}{\left|A|+|B|-|A\cap B\right|}$, where *A* and *B* are sets of AEs associated with drug *A* and drug *B*, respectively. For example, ipilimumab is associated with 100 AEs, and nivolumab is associated with 208 AEs. The number of overlapping terms between these two drugs is 57, and the Jaccard similarity is 0.229 [57/(10 057)].

### Classify extracted irAEs based on targeted organ systems and perform class enrichment analysis

To understand which organ systems were significantly affected by ICIs, we classified irAEs based on MedDRA classification scheme [18]. The MedDRA classification system is organized by the System Organ Classes (SOC), divided into High-Level Group Terms (HLGT), High-Level Terms (HLT), Preferred Terms (PT), and finally into LLT. We classified extracted irAEs into SOC. For instance, the 208 extracted irAEs for nivolumab were classified into 21 SOC classes, including “immune system,” “skin,” “metabolism,” and “endocrine.” For each SOC class, we assessed its probability of being associated with a ICI (or the set of irAEs associated with the ICI) as compared to its probability of being associated with the same number of randomly selected AEs. The random process was repeated 1000 times, and a t-test was used to assess the statistical significance. For example, among the 21 SOC classes associated with nivolumab, 9 were significantly enriched, including 4-fold enrichment for “immune system.” We then performed finer-grained classification based on the next-level HLT classification. For example, the 208 irAEs for nivolumab were classified into 87 HLT classes, including “autoimmune diseases,” “thyroid gland disorders,” and “heart failures.” Among the 87 HLT classes, 70 were significantly enriched, including 4.34-fold enrichment for the class “autoimmune disorders” and 5.3-fold enrichment for the class “thyroid gland disorders.”

### Identify broad-spectrum (nontarget-specific) and narrow-spectrum (target-specific) irAEs

To understand the relationships between irAEs and genetic targets of immune checkpoint inhibition, we identified irAEs that were shared among all six ICIs. We then identified target-specific irAEs for PD-L1/PD-1 inhibition and for CTLA-4 inhibition. The irAEs unique for PD-L1/PD-1 are irAES that were associated with all PD-L1/PD-1 inhibitors (atezolizumab, avelumab, durvalumab, nivolumab, pembrolizumab), but not with CTLA-4 inhibitor (ipilimumab). Similarly, we identified irAEs specific for CTLA-4, which were irAEs associated with CTLA-4 inhibitor but absent from PD-L1/PD-1 inhibitors.

## RESULTS

### Summary of extracted irAEs for six FDA-approved ICIs

We manually extracted irAEs from FDA drug labels and mapped extracted irAEs to unique preferred terms (PTs) based on the MedDRA terminology. As shown in Table 2, the majority (95%-96%) of irAE terms extracted from FDA drug labels can mapped to terms from MedDRA. The number of irAEs for six ICIs ranges from 100 for ipilimumab to 208 for nivolumab.

**Table 2.** Extracted irAEs for six FDA-approved immune checkpoint inhibitors

Name	Target	Total irAEs	Mapped irAEs	Mapping rate (%)
Atezolizumab	PD-L1	124	119	96.0
Avelumab		133	127	95.5
Durvalumab		108	103	95.4
Ipilimumab	CTLA-4	100	95	95.0
Nivolumab	PD-1	208	198	95.2
Pembrolizumab		191	182	95.3

The numbers of irAEs were not necessary correlated with the year the drugs were first approved. For example, ipilimumab was first approved in 2011 and was associated with 100 irAEs. Nivolumab was first approved in 2014 and was associated with 208 irAEs. Drugs with the same genetic targets tend to have similar numbers of irAEs as compared to drugs targeting different targets. The number of irAEs for three PD-L1 inhibitors ranges from 108 to 133, the number of irAEs for the two PD-1 inhibitors ranges from 191 to 208, and the number of irAEs for the CTLA-4 inhibitor is 100. The fact that ICIs with the same targets have similar number of irAEs than ICIs with different targets, indicates that certain irAEs may be caused by on-target effects of ICIs.

### Comparative analysis of irAE profiles

#### irAEs associated with PD-L1 inhibitors

**Figure 1.**
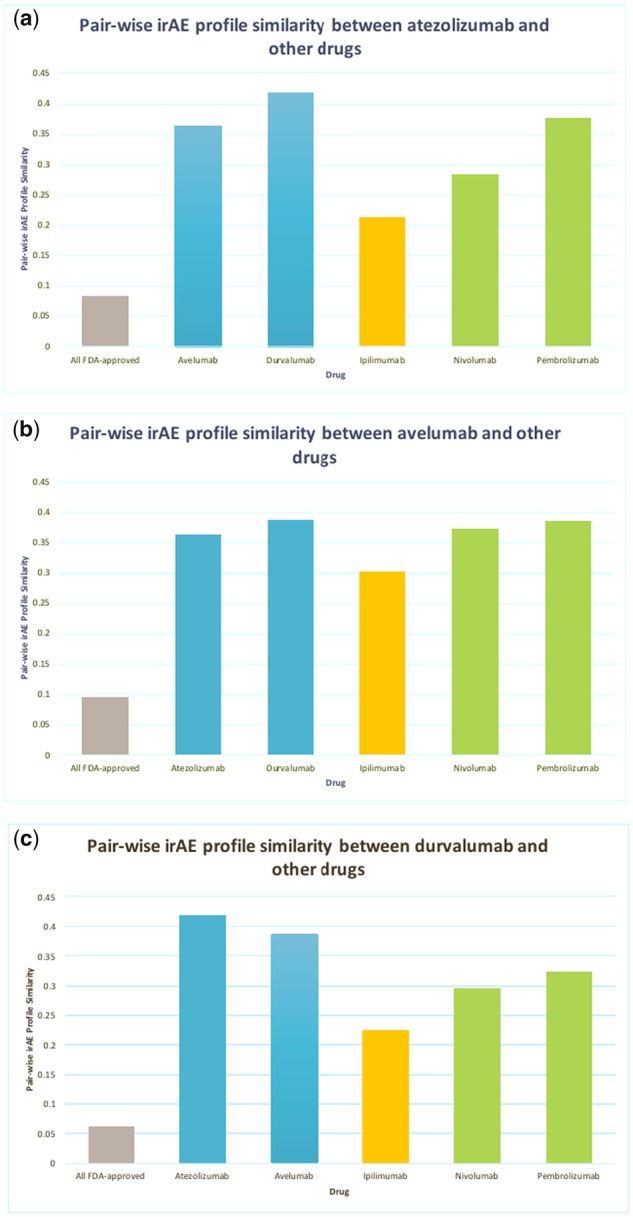
Pairwise irAE profile similarities between PD-L1 inhibitors (atezolizumab, avelumab, and durvalumab) and other drugs including 5 ICIs and 1507 FDA-approved non-ICI drugs (“All FDA-approved”). PD-L1 inhibitors (blue), PD-1 inhibitors (green), CTLA-4 inhibitor (orange), and other drugs (grey).

We compared the pairwise similarities of irAE profiles between PD-L1 inhibitors (atezolizumab, avelumab and durvalumab) and others drugs (other ICIs and 1507 FDA-approved drugs). As shown in Figure 1, PD-L1 inhibitors had more similar irAE profiles to other PD-L1 or PD-1 inhibitors than CTLA-4 inhibitor (ipilimumab). For example, the irAE profile similarity of atezolizumab is 0.43 to durvalumab (PD-L1 inhibitor), 0.21 to ipilimumab (CTLA-4 inhibitor) and 0.28 to nivolumab (PD-1 inhibitor). In addition, the similarities between PD-L1 inhibitors and other ICIs are significantly higher (ranging from 0.23 to 0.43) than those for 1507 FDA-approved non-ICI drugs (ranging from 0.06 to 0.09). However, the similarity among three PD-L1 inhibitors is not perfect (ranging from 0.36 to 0.43). Several explanations are possible for the imperfect similarity. First, the irAE profiles of ICIs may depend on the patient disease characteristics. These three PD-L1 inhibitors were approved to treat different cancer patients: atezolizumab for NSCLC, avelumab for metastatic Merkel cell carcinoma, and durvalumab for locally advanced or metastatic urothelial carcinoma. Second, cancer patient population on PD-L1 inhibitors may have different demographics, disease comorbidities, co-occurrent medications, among others. Third, not all irAEs have been captured in FDA drug labels and efforts are needed to extract more complete irAEs from other data resources such as published literature, patient electronic health records, and FDA postmarket surveillance system. Fourth, although these three ICIs have the same genetic on-target PD-L1, they may have different off-targets and some irAEs may be caused by drug’s off-target effects.

#### irAEs associated with CTLA-4 inhibitor

**Figure 2.**
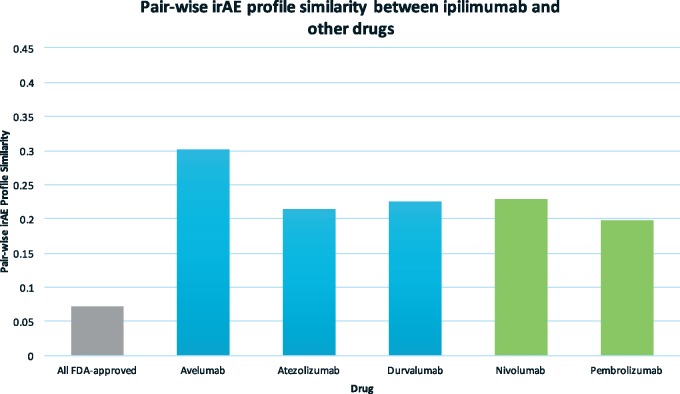
Pairwise irAE profile similarities between ipilimumab, the CTLA-4 inhibitor, and other drugs, including 5 ICIs and 1507 FDA-approved non-ICI drugs (“All FDA-approved”). PD-L1 inhibitors (blue), PD-1 inhibitors (green), CTLA-4 inhibitor (orange), and other drugs (grey).

The similarity between ipilimumab, a CTLA-4 inhibitor, and other ICIs ranges from 0.2 to 0.3 (Figure 2), which is lower than the similarities among the PD-L1/PD-1 inhibitors (ranging from 0.3 to 0.42) (Figure 1). This result indicates that some irAEs are caused by drug’s on-target effects (CTLA-4 or PD-L1/PD-1). The average similarity between ipilimumab and 1507 FDA-approved non-ICI drugs is 0.072, significantly lower than those among ICIs.

#### irAEs associated with PD-1 inhibitors

**Figure 3.**
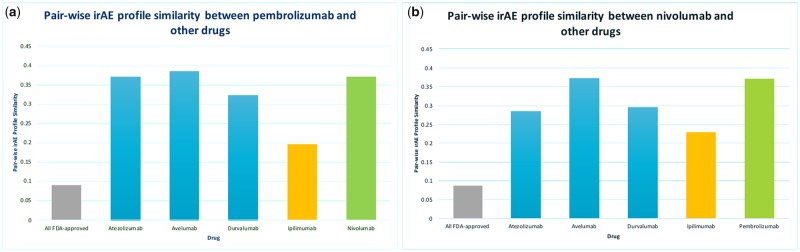
Pairwise irAE profile similarities between PD-1 inhibitors (pembrolizumab, nivolumab) and other drugs including 5 ICIs and 1507 FDA-approved non-ICI drugs (“All FDA-approved”).

Pembrolizumab and nivolumab are PD-1 inhibitors. The irAE profile of PD-1 inhibitors are more similar to other PD-1 or PD-L1 inhibitors (ranging from 0.28 to 0.38) than to ipilimumab (ranging from 0.19 to 0.22) (Figure 3). The average similarity between PD-1 inhibitors and 1507 FDA-approved non-ICI drugs is 0.08. Intriguingly, both pembrolizumab and nivolumab were first approved in the same year of 2014 for the treatment of advanced melanoma; however, their irAE profile similarity is only 0.36, suggesting that the majority of irAEs were caused by unknown off-targets of PD-1 inhibitors. The extracted irAEs when combined with existing genetic and genomic databases will set the foundation to develop data-driven computational approaches to understand both on- and off-target effects of ICIs.

### ICIs target a wide range of body systems

**Figure 4.**
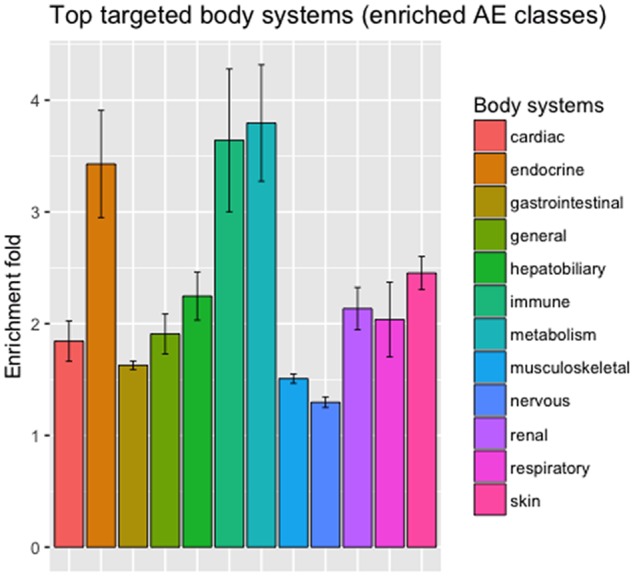
Enriched irAEs classes based on body systems. irAEs were classified based on MedDRA SOC-level classification.

We investigated which body systems were significantly targeted by ICIs. For each ICI, we classified its associated irAEs based on organ systems (SOC groups in MedDRA) and performed enrichment analysis to examine which categories of irAEs were significantly enriched. For example, the 100 irAEs associated with ipilimumab were classified into 23 SOC groups, among which 11 groups were significantly enriched, including 6-fold enrichment for the class “immune system disorders” (eg “autoimmune hepatitis,” “autoimmune thyroiditis,” “Guillain-Barre syndrome,” and “myasthenia gravis”). Figure 4 shows top body systems enriched for all six ICIs. Our analysis showed that ICIs significantly targeted a wide range of body systems, including “endocrine system,” “immune system,” and “metabolism.”

**Figure 5.**
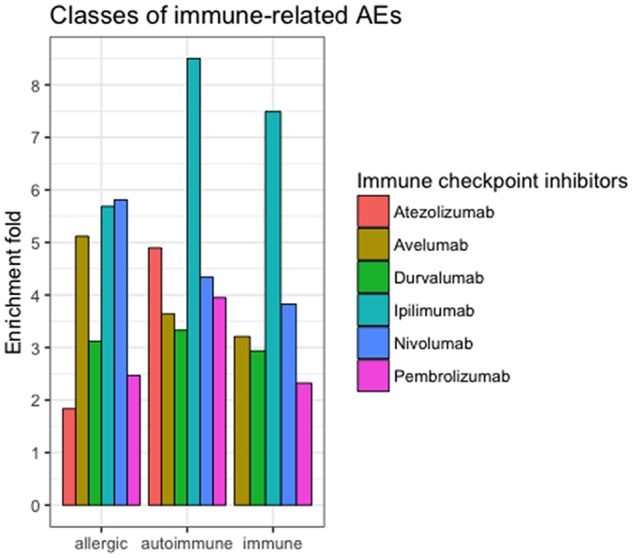
Immune-related subclasses of irAEs significantly enriched for six ICIs. Immune-related AEs were classified based on MedDRA HLT-level classification.

We performed finer-grained classification based on the next-level HLT classes. Figure 5 shows the enrichments of three immune-related subclasses (“allergic conditions,” “autoimmune disorders,” and “immune disorders”). For example, the 208 irAEs associated with nivolumab were classified into 87 HLT classes, including “autoimmune diseases,” “thyroid gland disorders,” and “heart failures.” Among the 87 HLT classes, 70 were significantly enriched, including the class “autoimmune disorders” (4.34-fold enrichment) and “thyroid gland disorders” (5.3-fold enrichment). Interestingly, ipilimumab was associated with significantly more “autoimmune diseases” (8.5-fold enrichment) and “immune disorders” (7.5-fold enrichment) as compared to other ICIs.

### Broad-spectrum (nontarget-specific) and narrow-spectrum (target-specific) irAEs

To understand the relationships between irAEs and immune checkpoint inhibition targets, we investigated irAEs shared among all ICIs. Table 3 shows 21 common irAEs. These shared irAEs may be caused by the class effects of immune checkpoint inhibition. We then identified target-specific irAEs for PD-L1/PD-1 inhibition and for CTLA-4 inhibition. A total of 20 irAEs are unique for PD-L1/PD-1 inhibitors and 28 were unique for CTLA-4 inhibitor (Table 4). For example, Type 1 diabetes was associated with all five PD-L1/PD-1 inhibitors, but not associated with ipilimumab, indicating that PD-L1/PD-1 inhibition but not CTLA-4 inhibition may be involved in the disease mechanisms of Type 1 diabetes. Note that our current analysis is based on irAEs reported in FDA drug labels. As more complete irAEs will be reported in FDA drug labels or other data resources, the lists of common or specific irAEs may change.

**Table 3.** 21 irAEs that are shared among all six ICIs

	AEs shared among six ICIs	
Abdominal pain	Abortion	Adrenal insufficiency
Alanine aminotransferase	Aspartate aminotransferase	Asthenia
Blood alkaline phosphatase	Colitis	Death
Decreased appetite	Diarrhea	Fatigue
Hepatitis	Hyperthyroidism	Hypophysitis
Hypothyroidism	Infusion reaction	Nausea
Pneumonitis	Pyrexia	Rash

**Table 4.** 10 irAEs unique for PD-L1/PD-1 inhibitors and 10 irAEs unique for CTLA-4 inhibitor

Unique for PD-L1/PD-1 inhibitors	Unique for CTLA-4 inhibitor
(Total = 20)	(Total = 28)
Acute kidney injury	Acute respiratory distress syndrome
Dermatitis acneiform	Adrenocortical insufficiency acute
Embryo-fetal toxicity	Angiopathy
Hyperglycemia	Blepharitis
Hyponatremia	Cushing’s syndrome
Lymphopenia	Endocrine ophthalmopathy
Rash maculopapular	Hypogonadism
Rash pruritic	Mental status changes
Type 1 diabetes mellitus	Neurosensory hypoacusis
Urinary tract infection	Pericarditis

## DISCUSSION AND CONCLUSION

In this study, we manually extracted irAEs for six FDA-approved ICIs from FDA drug labels, performed comparative analysis of irAE profiles among ICIs and 1506 FDA-approved drugs, and investigated which organ systems were significantly targeted by ICIs. The extracted irAEs can serve as the gold standard to evaluate automatic irAE extractions from other data resources and set a foundation to develop computational approaches to understand biological mechanisms of irAEs. To the best our knowledge, our study presents the first effort towards building a highly accurate, standardized irAE data resource and performed comparative analysis to characterize and understanding extracted irAEs. We have made the data publicly available at: http://nlp.case.edu/public/data/irAEs_FDA.

Future efforts are needed for comprehensively characterizing and understanding irAEs in cancer patients. First, it will be necessary to extract irAEs from other data resources, such as published biomedical literature, clinical trial reports, FDA postmarket drug safety surveillance systems, patient electronic health records, among others. It is known that FDA drug labels only capture a small portion of drug side effects.^10^^,^^16^^,^^20^ To extract irAEs from other data resources, which are in much large scale than FDA drug labels, automatic approaches such as natural language processing, data mining, and machine learning techniques will be necessary.

Second, not all patients taking ICIs will develop the same irAEs, therefore it is necessary to extract patient-level irAEs, which may be affected by patient genetics, disease characteristics, demographics, co-occurrent drugs, among others. These patient-level irAEs are important for tailored personalized ICI treatments and irAE management. Unlike FDA drug labels, many other data resources contain rich information of patient population information, such as patient electronic health records, FDA-post-market surveillance system and published biomedical literature.

Third, our study demonstrated that the majority of irAEs were not caused by drugs’ on-target effects, therefore it is important to identify and understanding how off-targets of ICIs are involved in irAEs.

## FUNDING

This work was supported by the NIH Director’s New Innovator Award under the Eunice Kennedy Shriver National Institute Of Child Health & Human Development of the National Institutes of Health (DP2HD084068); NIH National Institute of Aging (1 R01 AG057557-01); American Cancer Society Research Scholar (RSG-16-049-01 – MPC); NIH Clinical and Translational Science Collaborative of Cleveland (1UL1TR002548-01).

*Conflict of interest statement*. None declared.

## CONTRIBUTORS

Xu and Wang have jointly conceived the idea, designed and implemented the algorithms and prepared the manuscript. All authors read and approved the final manuscript.
